# P249: Serratia marcescens among neonates

**DOI:** 10.1186/2047-2994-2-S1-P249

**Published:** 2013-06-20

**Authors:** EB Brusina, I Turgeneva, V Borisov

**Affiliations:** 1 Department of Epidemiology, Kemerovo State Medical Academy, Kemerovo, Russian Federation; 2 Laboratory of Ultrastructural Methods of Tissue Investigation, Research Institute for Complex Issues of Cardiovascular Diseases under the Siberian Branch of the Russian Academy of Medical Sciences, Kemerovo, Russian Federation

## Introduction

Healthcare-associated infections (HAIs) remain a permanent challenge among neonates. *Serratia marcescens* (SM) is a rare causative agent of HAIs, responsible for not more than 2% of HAI cases.

## Objectives

To analyze a colonization of SM in a neonatal intensive care unit (NICU).

## Methods

We carried out an observational study among 489 neonates in a NICU, which included microbiological and epidemiological investigations during the period from March till October, 2012. We performed pulsed field gel electrophoresis (PFGE), random amplification of polymorphic DNA, and we also conducted a genotyping to reveal high pathogenicity island (HPI) and genes encoding haemolysin A (ShlA), haemolysin B (ShlB), and phospholipase A (PLA). In addition, we performed antimicrobial susceptibility testing and scanning electron microscopy (SEM).

## Results

We found that 72 neonates were colonized by SM without any cases of acute infection. PFGE revealed that only one SM strain was responsible for this colonization. SEM did not reveal any features of the pathogen, and inoculated SM strain was susceptible to imipenem and cefoperazone sulbactam only. Out of 41 cases investigated, 12 possessed ShlA gene, and no HPI, ShlB and PLA genes were determined. All bacteria belonged to RAPD type 1. Nebulizer was recognized as a source of SM.

## Conclusion

SM was revealed during the pre-epidemic situation and was successfully eliminated after additional chemical disinfection of nebulizers. Our data shed light on transmission of SM-caused HAIs.

## Disclosure of interest

None declared

